# Liver X Receptors Protect Dorsal Root Ganglia from Obesity-Induced Endoplasmic Reticulum Stress and Mechanical Allodynia

**DOI:** 10.1016/j.celrep.2018.09.046

**Published:** 2018-10-09

**Authors:** Chaitanya K. Gavini, Angie L. Bookout, Raiza Bonomo, Laurent Gautron, Syann Lee, Virginie Mansuy-Aubert

**Affiliations:** 1 Cell and Molecular Physiology, Stritch School of Medicine, Loyola University Chicago, Maywood, IL 60153, USA; 2 Division of Hypothalamic Research, Department of Internal Medicine, University of Texas Southwestern Medical Center, Dallas, TX 75390, USA; 3 Department of Pharmacology, University of Texas Southwestern Medical Center, Dallas, TX 75390, USA; 4 Lead Contact

## Abstract

Obesity is associated with many complications, including type 2 diabetes and painful neuropathy. There is no cure or prevention for obesity-induced pain, and the neurobiology underlying the onset of the disease is still obscure. In this study, we observe that western diet (WD)-fed mice developed early allodynia with an increase of ER stress markers in the sensory neurons of the dorsal root ganglia (DRG). Using cell-specific approaches, we demonstrate that neuronal liver X receptor (LXR) activation delays ER stress and allodynia in WD-fed mice. Our findings suggest that lipid-binding nuclear receptors expressed in the sensory neurons of the DRG play a role in the onset of obesity-induced hypersensitivity. The LXR and lipid-sensor pathways represent a research avenue to identify targets to prevent debilitating complications affecting the peripheral nerve system in obesity.

## In Brief

The mechanism underlying obesityinduced pain is explored by Gavini et al. using cell-specific models. Their analysis reveals that in sensory neurons of the dorsal root ganglia, LXR activation delays western diet-induced ER stress and allodynia. These findings suggest that LXRs in sensory neurons are involved in nociception induced by western diet nutrition.

## Graphical Abstract

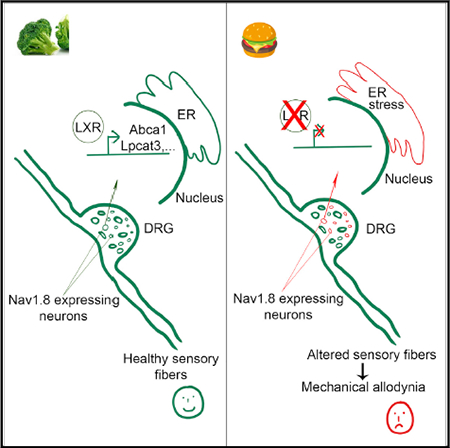

## INTRODUCTION

Obesity, which has reached epidemic proportions in the United States and is increasing worldwide, is associated with type 2 dia-betes, dyslipidemias, cardiovascular pathologies, and neurodegenerative disorders (Flegal et al., 2016; Schwartz et al., 2017; Hotamisligil, 2006). This constellation of symptoms, termed metabolic syndrome, continues to rise, particularly in countries adopting a westernized diet (International Diabetes Federation, 2014). More than half of patients with diabetes, alone or in combination with other components of metabolic syndrome, develop some form of peripheral neuropathy (Feldman et al., 2017; O’Brien et al., 2014a). The pathophysiology of diabetic neuropathy is complex and under debate. There is a recent body of evidence linking painful neuropathy to obesity, independent of diabetes, highlighting the importance of lipid metabolism in the onset of neuropathy (Guilford et al., 2011; O’Brien et al., 2014b). Because of this complexity, there are no pharmacological treatments available that prevent or cure obesity-induced pain, and understanding the early mechanisms is critical for developing therapeutic prevention.

One such mechanism involves the endoplasmic reticulum (ER), the organelle responsible for protein folding and trafficking. When the ER becomes stressed because of the accumulation of unfolded proteins, the unfolded protein response (UPR) is activated (Ron and Walter, 2007). The UPR regulates synthesis of lipids and protein components of the ER to meet varying demands on protein folding in response to pathophysiological conditions (Schuck et al., 2009). The ER is also the major site for the synthesis of sterols and phospholipids and regulates membrane lipid homeostasis (Fagone and Jackowski, 2009). It has been previously reported that obesity rapidly induces ER stress in various tissues, including neurons, which in the long term leads to insulin resistance and type 2 diabetes (Ozcan et al., 2004; Williams et al., 2014). Current evidence describes ER stress in neurons of the peripheral nervous system (PNS) as a potential mechanism in diabetic neuropathy (O’Brien et al., 2014a; Lupachyk et al., 2013; Inceoglu et al., 2015), suggesting that early modulation of ER stress in the PNS could prevent obesity-induced hypersensitivity (Biessels et al., 2014).

The liver X receptors (LXRs, LXRα, and LXRβ) are lipid nuclear receptors and play a crucial role in the regulation of cholesterol and fatty acid homeostasis (Hong and Tontonoz, 2008). Although well characterized in metabolic tissues, in which they regulate lipid homeostasis (Kalaany and Mangelsdorf, 2006), membrane phospholipids, and inflammation (Rong et al., 2013), their cell-specific role in the PNS remains to be clarified. The present study shows that LXR agonist treatment prevents progression of obesity-induced allodynia. Using LXR agonist and cell-specific models, we provide insights into the cellular and molecular pathogenesis of obesity-associated allodynia and link LXRs with ER stress in dorsal root ganglia (DRG) neurons.

## RESULTS AND DISCUSSION

### The Nuclear Receptors LXRs Are Transcriptionally Active in the DRG and Protect from Saturated Fatty Acid-Induced ER Stress

**Figure 1.**
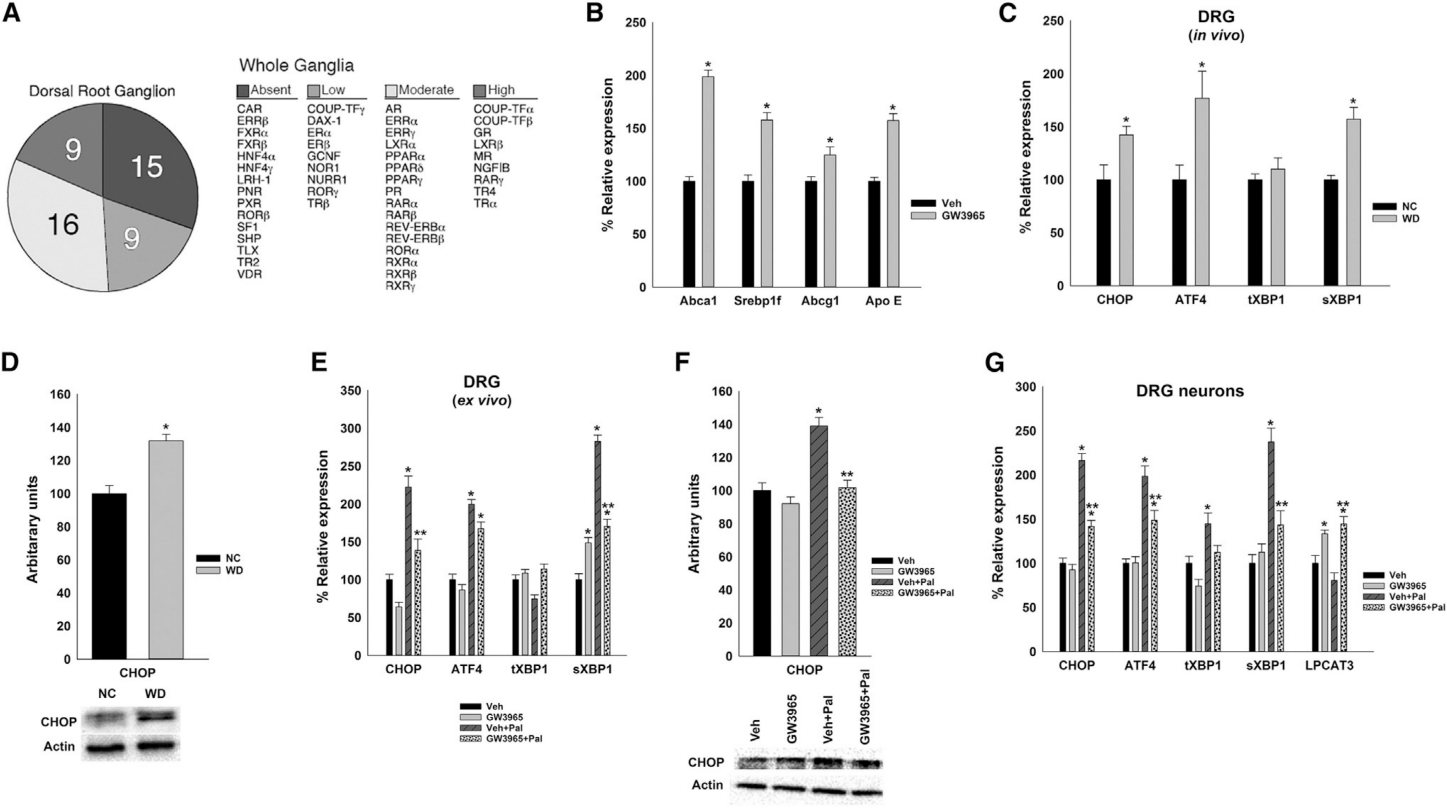
LXR Agonist (GW3965) Regulates DRG Gene Expression and Protects from Palmitate-Induced ER Stress in DRG (A) Distribution of nuclear receptor mRNA in whole dorsal root ganglia (DRG). (B) LXR agonist increases gene expression of LXR targets in organotypic cultures of DRG. (D and F) Protein levels of CHOP, an ER stress marker, in DRG of mice fed WD compared to NC (D) (n = 10 DRG/group) and in *ex vivo* organotypic whole DRG cultures treated with palmitate and LXR agonist (F) (n = 3 individual experiments, n = 5 or 6 DRG/group). (C, E, and G) mRNA levels of ER stress markers, in DRG of WD and NC fed mice (n = 8 mice/group) (C), in organotypic whole DRG cultures (E), and in primary neuronal culture of DRG neurons treated with LXR agonist and palmitate (G) (n = 5 individual experiments). All values are mean ± SEM, with vehicle group defined as 100%. *p < 0.05 with vehicle, **p < 0.05 with vehicle + palmitate.

Nuclear receptors (NRs) are transcription factors that bind to lipophilic hormones and dietary-derived lipids to regulate meta-bolic, inflammatory, and oxidative pathways (Chawla et al., 2001). A high-throughput real-time PCR screen was performed to investigate the expression pattern of the 49 murine NRs in the DRG of wild-type (WT) mice as previously shown (Bookout et al., 2006, 2013). NRs were classified according to their expression levels and by physiological relevance (Figure 1A). The analysis of the data showed that several NRs important in lipid homeostasis and inflammation were expressed at moderate to high levels in the DRG, including LXRs. Using *in situ* hybridization, we confirmed the expression of LXRs in the DRG neurons (not shown).

Because LXRs are important regulators of lipid metabolism in many cell types (Hong and Tontonoz, 2008; Kalaany and Mangelsdorf, 2006), we hypothesized that the LXR pathway may mediate certain aspects of cholesterol and lipid remodeling in the DRG. We observed significant increase of LXR canonical gene expression, such as ATP-binding cassette transporter (ABCA1), sterol regulatory element binding transcription factor 1f (SREBP1F), and apolipoprotein E (APO E), in DRG explants stimulated with GW3965 (LXR agonist) (Figure 1B), confirming that LXRs are present and transcriptionally active in the DRG.

Early activation of ER stress pathways has been identified in type 1 and type 2 diabetes (Lupachyk et al., 2013). Increased expression of the ER stress marker C/EBP homologous protein (CHOP) was reported in metabolic tissues of diabetic mice, while targeted disruption of chop gene delayed the onset of diabetes (Oyadomari et al., 2002). We identified an early upregulation in ER stress markers in the DRG of western diet (WD)-fed mice compared with control mice (Figures 1C and1D). Compared with NC-fed mice, WD-fed mice had higher mRNA levels of chop, activating transcription factor 4 (atf4), and spliced variant of X-box binding protein-1 (sxbp1) expression in the DRG (Figure 1C). Lipid overload, particularly saturated fatty acids such as palmitate, triggers the UPR (Borradaile et al., 2006; Volmer et al., 2013; Cragle and Baldini, 2014; Rong et al., 2013). Palmitate stimulation of DRG explants also increased the levels of chop and atf4 and also increased the formation of sxbp1 (Figure 1E), which is involved in enhancing the folding capacity of the ER to minimize ER stress (Hotamisligil, 2010). Recently, activation of LXRs has been shown to decrease lipo-toxicity, suppressing palmitate-induced UPR signaling in the liver (Rong et al., 2013). We hypothesize that LXRs could modulate lipid-induced ER stress in the DRG neurons.

Compared with vehicle, GW3965 treatment decreased the mRNA levels of ER stress markers in palmitate-treated DRG explants (Figure 1E). Similar results were obtained when DRG primary neurons were treated with palmitate and GW3965 (Figure 1G). These findings suggest that in DRG neurons (but also in other cell types in the ganglia), LXRs could modulate saturated fatty acid-induced ER modification. Rong et al. (2013) showed that LXRs could regulate the expression of LPCAT3 (lysophospholipid acyltransferase). Interestingly, we also observed a significant increase of lpcat3 mRNA in dissociated pure DRG neurons stimulated with LXR agonist (Figure 1G), suggesting that LPCAT3 is also a target of LXR in DRG neurons.

### LXRs Agonist Treatment Delays WD-Induced Allodynia

WT mice were maintained on a standard diet (normal chow [NC]) or WD (high fat, high sucrose, high cholesterol) for 12 weeks. As previously shown in Mansuy-Aubert et al. (2015), WD-fed mice weighed significantly more after 5 weeks of WD (Figure S1A). WD-fed mice had significantly higher levels of circulating leptin and insulin (Figures S1F and S1G) and showed impaired insulin sensitivity (Figures S1B and S1C). Compared with NC-fed mice, WD-fed mice also had higher levels of serum triglycerides and cholesterol (Figures S1D and S1E). Compared with NC livers, livers of mice on WD also showed higher fat accumulation (Figures S1K and S1L). The mechanical hypersensitivity observed early in peripheral neuropathy is associated with meta-bolic syndrome and independent of diabetes (Guilford et al., 2011; O’Brien et al., 2014b), suggesting that the WD-fed model (obese and glucose intolerant) may represent an appropriate model to study the early onset of obesity-induced peripheral neuropathy. However, it is important to stress that although sensory hypersensitivity is one of the hallmarks of neuropathy (Biessels et al., 2014) and is observed early in neuropathy phenotype, it does not always represent an early neuropathy event. Indeed, we cannot exclude that hypersensitivity could also be observed in absence of further neuropathy in obesity models.

We performed the von Frey test in WD-and NC-fed mice. Compared with NC mice, WD mice had a lower threshold (Figure S1H), suggesting an increased sensitivity to innocuous stimuli. Thermal sensitivity had also been reported in diabetic neuropathy, but the thermal nociception phenotype is unclear in a diet-induced obesity model (O’Brien et al., 2014b). These sensory behaviors involve some overlapping and independent complex circuitries (Lolignier et al., 2015). We evaluated the thermal nociception of NC- versus WD-fed mice and observed that WD mice had a lower withdraw latency (Figure S1I), suggesting WD-induced hyperalgesia. Later stage neuropathy is defined by behavioral changes accompanied with anatomical changes (Biessels et al., 2014). We evaluated neuronal loss together with fiber morphology, and no change was observed until 16 weeks of WD feeding compared with control (not shown). These observations were consistent with reports showing hypersensitivity preceding structural changes in diet-induced obesity and diabetes (Calcutt et al., 1996; Jayaraj et al., 2018; O’Brien et al., 2014b).

**Figure 2.**
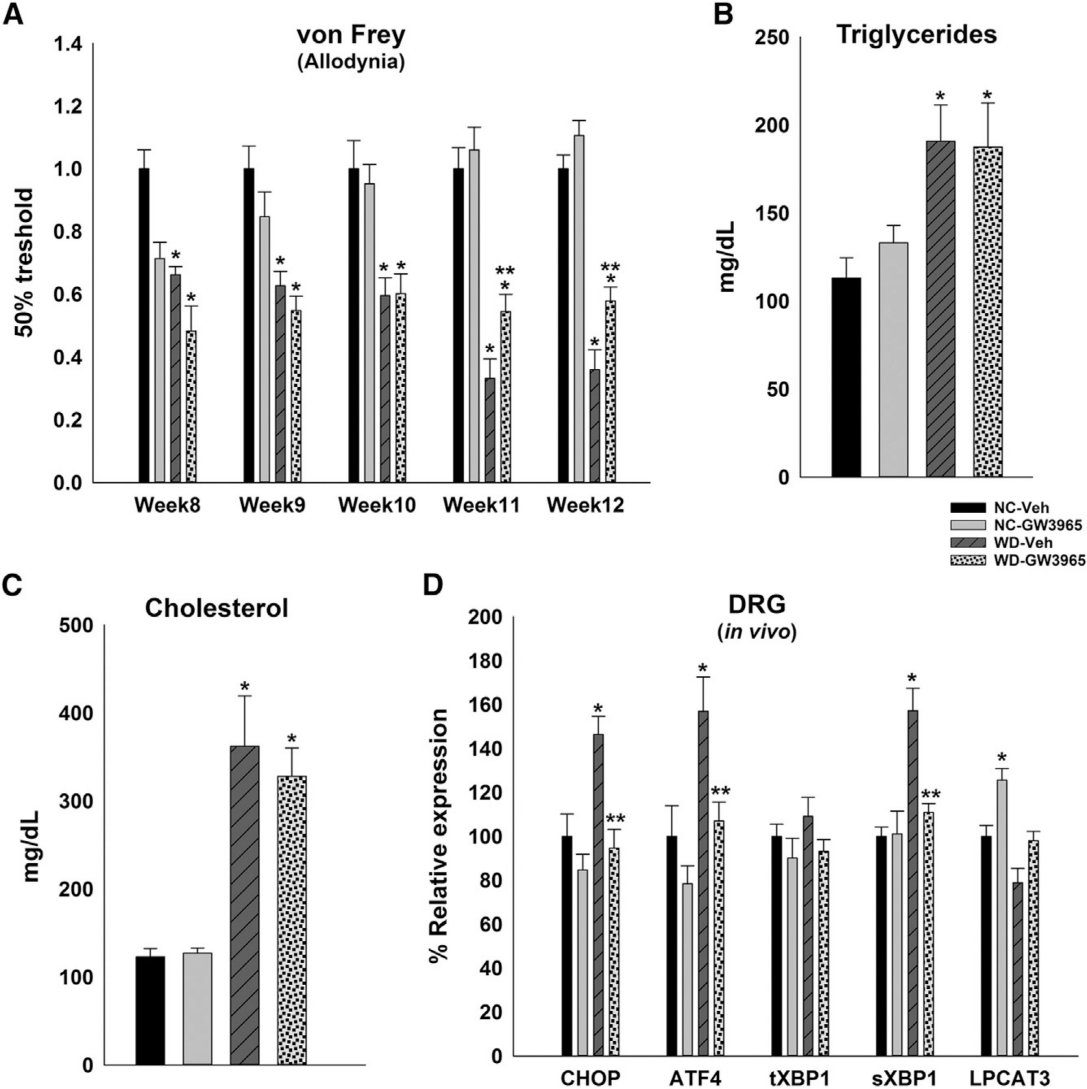
LXR Agonist (GW3965) Delays the Progression of Western Diet-Induced Allo-dynia and Protects the DRG from ER Stress (A) von Frey test to assess sensitivity of mice on either diet treated with LXR agonist to innocuous stimuli (e.g., week 8 = baseline, 8 weeks on WD; week 9 = 1 week after agonist admission, 9 weeks on WD). (B and C) Endpoint levels of serum triglycerides (B) and cholesterol (C) in mice fed NC or WD treated with agonist. (D) mRNA levels of ER stress markers in DRG of NC- or WD-fed mice treated with LXR agonist. n = 8 mice/group. All values are mean ± SEM. For mRNA, relative levels were plotted with NC-vehicle (Veh) group defined as 100%. *p < 0.05 with NC-Veh, **p < 0.05 with WD-Veh. See also Figure S1.

We then assessed whether sustained activation of LXRs could change the WD-induced allodynia. WT mice were fed either NC or WD while assessing allodynia. WD-fed mice start exhibiting hypersensitivity within 5 weeks on WD, reaching a significant difference by week 8 of WD (Figure 2A). Mice were treated for 3 weeks with either GW3965 (25 mg/kg body weight [BW]) or vehicle after 8 weeks of WD diet (12 weeks of age). As activation of LXRs elevates triglyceride levels in liver and plasma (Zelcer and Tontonoz, 2006), the dosage of LXR agonist was adjusted as previously shown (Mitro et al., 2007) (Figures 2B and2C).

We also verified in separate studies that the dosing scheme led to a sustained increase in DRG abca1 mRNA *in vivo* (not shown). Compared with vehicle treatment, LXR agonist treatment prevented the development of hypersensitivity overtime (Figure 2A). Then, we compared the expression of UPR target genes in the whole DRG of NC- or WD-fed mice treated with vehicle or GW3965. Activation of LXRs in WD-fed mice had decreased expression of ER stress markers (Figure 2D). These findings suggest that *in vivo* LXR activation can protect DRG cells against WD-induced ER stress to potentially prevent WD-induced allodynia.

### LXRs Suppress Saturated Fatty Acid- and WD-Induced ER Stress in Sensory Neurons

**Figure 3.**
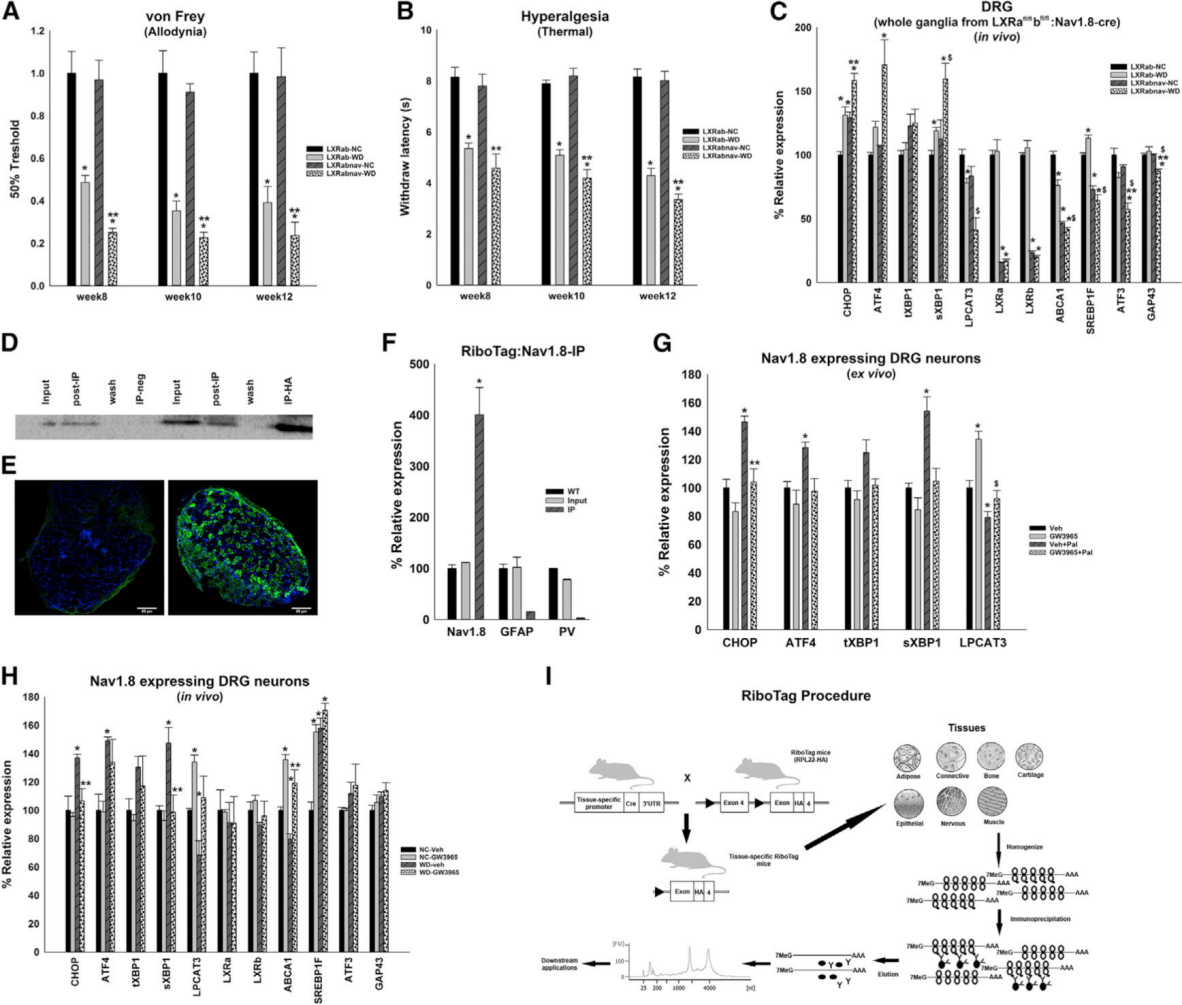
LXR Agonist Decreases Lipid-Induced ER Stress in DRG Neurons Expressing Nav1.8 (A) von Frey test to assess the sensitivity of LXRab and LXRabnav mice on either diet to innocuous stimuli (n = 8/group). *p < 0.05 compared with LXRab NC,**p < 0.05 compared with LXRabnav. (B) Thermal sensitivity test (Hargreaves method) to assess thermal nociception of LXRab and LXRabnav mice on either diet (n = 8/group). *p < 0.05 compared with LXRab NC, **p < 0.05 compared with LXRabnav. (C) mRNA levels of ER stress markers and canonical LXR pathway in DRG of NC- or WD-fed LXRab and LXRabnav mice. n = 8/group, with LXRab-NC group defined as 100%. *p < 0.05 compared with LXRab NC, **p < 0.05 compared with LXRabnav, ^$^p < 0.05 compared with LXRab-WD). (D) Western blot on whole DRG of RiboTag-Nav1.8-Cre mice after immunoprecipitation using anti-HA antibody. (E) Immunohistochemistry on DRG slices for HA in sensory neurons (green, HA; blue, DAPI/nuclei) (scale bar, 50 μm). (F) mRNA levels of positive (Nav1.8) and negative (GFAP, PV) markers of Nav1.8-expressing neurons in whole DRG (WT), input, and IP samples (n = 3 mice/group,n = 6–8 DRG/mice). (G) mRNA levels of ER stress markers, in sensory neurons from Ribotag-Nav1.8-Cre mice treated with LXR agonist and palmitate (*ex vivo*) (n = 3 individual experiments, n = 5 or 6 DRG/group). Vehicle group defined as 100%. *p < 0.05 with vehicle, **p < 0.05 with vehicle + palmitate, ^$^p < 0.05 compared with GW3965. (H) mRNA levels of ER stress markers and targets of LXR pathway in sensory neurons (*in vivo*) from NC- or WD-fed DRG of Ribotag-Nav1.8-Cre mice treated with GW3965 or vehicle. n = 8/group, with NC-vehicle group defined as 100%. *p < 0.05 with NC-vehicle, **p < 0.05 with WD-vehicle. (I) Generation of tissue-specific RiboTag mouse. We used sensory neuron-specific (Nav1.8) Cre mice to generate Ribotag-Nav1.8-Cre mice. All values are mean ± SEM. See also Figure S2.

Our data suggest that LXRs regulate diet-induced ER stress in the DRG. The DRG includes many cell types (neurons, Schwann cells, immune cells, etc.). To understand the cellular neurobiology underlying early allodynia induced by WD, we used cell-specific approaches. Nav1.8 is a tetrodotoxin-resistant sodium channel expressed in nociceptive neurons with small and medium-sized soma diameters located in the DRG and in the nodose ganglia (Gautron et al., 2011). Nav1.8 expressed in the DRG is involved in pain (Lai et al., 2002; Ruangsri et al., 2011; Gautron et al., 2011) and is targeted in painful type 2 diabetic neuropathy (Feldman et al., 2017). To evaluate the effect of saturated fatty acids and LXRs on sensory neurons of the DRG, we generated the sensory neuron-specific deletion of LXRs (LXRα and LXRβ) (LXRα^fl/fl^β^fl/fl^:Nav1.8Cre+/–; LXRabnav) by crossing LXRα^fl/fl^β^fl/fl^ (LXRab) mice with Nav1.8Cre+/– mice. Interestingly, loss of LXRα and LXRβ in sensory neurons expressing Nav1.8 further augmented WD-induced allodynia (Figure 3A) and hyperalgesia (Figure 3B), indicating that LXRs in the sensory neurons regulate WD-induced mechanical allodynia and hyperalgesia. Of note, the data presented in Figure 2A showed that LXR agonist prevent further WD-driven increase in allodynia occurring at weeks 10–11. We cannot exclude the possibility that the timely differences between the tissue-specific LXR deletion and the LXR agonist injected mice are due to unique pathways. Indeed, previous studies have demonstrated that gene expression response to synthetic LXR agonists could be different from the response to endogenous cholesterol-derived LXR ligands (Magida and Evans, 2018; Muse et al., 2018).

Although LXRab and LXRabnav mice weighed significantly more than control mice when fed WD (Figure S2A), WD-fed LXRabnav mice gained significantly less weight than their LXRab counterparts (Figure S2A). These data are consistent with previous reports showing a role of LXRs in Nav1.8-expressing neurons in control of BW (Mansuy-Aubert et al., 2015). As mentioned, Nav1.8 is expressed in the DRG and in the nodose ganglia of the vagus nerve (Mansuy-Aubert et al., 2015). To evaluate whether BW and metabolic changes may indirectly affect hypersensitivity, we generated mice lacking LXR in Phox2b-expressing neurons by crossing LXR floxed mice with Phox2bcre mice (Liu et al., 2014). Phox2b is a transcription factor expressed in the nodose ganglia neurons and in the brainstem but not in the DRG (Gautron et al., 2011; Liu et al., 2014).

LXRab and LXRabPhox2b mice were fed either WD or NC and assessed for the onset and progression of mechanical allodynia together with BW and energy balance. The loss of LXR in neurons expressing phox2b also decreased BW of WD-fed mice (Figure S2B), but the WD-induced allodynia was not exacerbated in mice lacking LXR in phox2b neurons (compared with control littermates; Figure S2C). Compared with their controls, no difference was found in either LXRabnav or LXRabphox2b mice with respect to glucose tolerance on either diet (not shown). These data suggest that LXR expressed in the Nav1.8 neurons located in DRG are responsible for the allodynia phenotype. In addition, these data suggest that most likely, the metabolic phenotype observed in the LXRabnav mice WD fed for 12 weeks has no indirect impact on mechanical sensitivity.

Loss of LXR in sensory neurons of the DRG decreases LXR canonical gene expressions (Figure 3C). We also observe an increased the ER stress pathways in the DRG of WD-fed mice lacking LXR in Nav1.8-expressing neurons (Figure 3C).

To study cell-specific pathways, we generated mice expressing an HA-tagged ribosomal protein (RPL22-HA) in the sensory neurons (RiboTag+/+:Nav1.8Cre+/–; RiboTag-Nav) by crossing RiboTag mice with hemizygous Nav1.8-Cre mice (RiboTag mice procedure; Figure 3I). *Ex vivo* DRG organotypic cultures of WT and RiboTag-Nav were treated with palmitate and GW3965 as described above. Sensory neuron-specific mRNAs were isolated from DRG of RiboTag-Nav as detailed in the STAR Methods. Bioanalyzer traces (Figure S2E) show the purity and integrity of mRNA isolated from immunoprecipitation (IP) of poly-somes from RiboTag-Nav. We verified (1) the presence of HA in the IP sample versus controls (Figure 3D) and (2) the presence of HA staining in Nav1.8-expressing neurons of the DRG (Figure 3E). We further evaluated the expression of a positive control gene (Scn10a/Nav1.8) and negative control genes (glial fibrillary acidic protein [gfap], parvalbumin [pv]) (Figure 3F). These data confirm that we have enriched mRNA from DRG Nav1.8-positive neurons.

The mRNA levels of ER stress markers undergoing translation in Nav1.8 neurons were analyzed. Chop, atf4, and sxbp1 mRNA levels were increased in sensory neurons treated with palmitate compared with vehicle controls (Figure 3G). These increases were reduced by treating with GW3965 (Figure 3G), suggesting that LXRs regulate saturated lipid-induced ER stress in the DRG expressing Nav1.8 neurons.

To evaluate the effect of LXR on DRG sensory neurons *in vivo*, RiboTag-Nav mice fed NC or WD were injected with GW3965 as described above. Three weeks after injection, mRNAs were isolated from DRG of RiboTag-Nav mice by IP as described in the STAR Methods. The mRNA level of LXR targets were evaluated. We observed increases in abca1, srebp1f, and lpcat3, suggesting that LXRs regulate lipid metabolism in DRG sensory neurons *in vivo*. The mRNA levels of ER stress markers in translation were also analyzed, and we observed that the WD increases in chop and sxbp1 mRNA were significantly decreased following GW3965 injection (Figure 3H). These data showed that LXRs may regulate lipid metabolism in sensory neurons of the DRG *in vivo*. The results also showed that GW3965 injection *in vivo* partially blunts the WD-induced ER stress in sensory neurons of the DRG. Our data suggest that regulation of lpcat3 and/or abca1 might drive WD-induced ER stress in sensory neurons. Of note, however, the *in vitro* data presented in Figures 2D and3G seems to argue against an LPCAT3-driven mechanism. These slight discrepancies between *in vitro* and *in vivo* data could be explained by the use of two different methodologies, and future investigation studying LXR pathways in LPCAT3 knockout would be necessary to conclude about an LXR/ lpcat3/ER stress pathway in neurons. Abca1 represent a candidate that could drive LXR-dependent ER stress in DRG neurons. Abca1 regulates cholesterol efflux and is involved in lipid raft formation and ER stress (Koseki et al., 2007; Landry et al., 2006). Cholesterol and phospholipids are crucial for lipid raft formation and ion channel clustering that regulates nerve excitability (Boslem et al., 2013; Pristerà et al., 2012; Tsui-Pierchala et al., 2002). Any decrease in abca1 expression may change the fiber’s membrane lipid rafts and may lead to altered neuronal sensitivity.

Altogether, our data indicate that LXRs are active in the sensory neurons of the DRG, where they regulate WD-mediated ER stress and WD-induced early allodynia. Further investigation would be necessary to better delineate whether LPCAT3 or others LXR targets regulating cholesterol metabolism (such as abca1) account for the decrease in ER stress associated with the decreased progression of allodynia observed after LXR agonist injection. More PNS cell-specific studies would be helpful to better understand the complex obesity-induced neuropathy disease. They will also certainly advance our knowledge of the tissue-specific function of the broadly expressed lipid NRs.

## STAR★METHODS

### KEY RESOURCES TABLE

**Table:** 

REAGENT or RESOURCE	SOURCE	IDENTIFIER
Antibodies		
Mouse monoclonal anti-HA	Biolegend	Cat# 901513; RRID:AB_291262
Goat Anti-mouse 488	Abcam	ab150113; RRID:AB_2576208
Mouse monoclonal anti-beta actin	Abcam	ab8226; RRID:AB_306371
Mouse monoclonal anti-DDIT3 (CHOP)	Abcam	ab11419; RRID:AB_298023
Rat anti-HA-peroxidase	Sigma-Aldrich	#12013819001; RRID:AB_390917
Chemicals, Peptides, and Recombinant Proteins		
LXR agonist; GW3965	Axon Medchem	Axon 1266
Critical Commercial Assays		
AlphaTrak glucometer strips	Fisher Scientific	NC0505524
Total serum triglycerides	Fisher Scientific	TR22421
AlphaTrak glucometer for rodents	Fisher Scientific	NC0499130
Total serum cholesterol	Fisher Scientific	TR13421
Insulin Elisa kit	EMD Millipore	EZRMI-13K
Acturus PicoPure RNA extraction kit	Applied Biosystems	KIT0204
RNeasy Micro Kit	QIAGEN	Cat# 74004
Quant-iT RIboGreen RNA assay kit	Invitrogen	R11490
FastStart Universal SYBR Green Master	Roche Life Science	4913914001
TaqMan Gene Expression Master Mix	Applied Biosystems	4369016
Leptin Elisa kit	EMD Millipore	EZML-82K
Experimental Models: Organisms/Strains		
Mouse: C57BL/6J	Jackson Laboratory	Jax: 000664
Mouse: RiboTag	Jackson Laboratory	Jax: 011029
Mouse: Nav1.8Cre	UT Southwestern	Mansuy-Aubert et al., 2015
Mouse: LXRa^fl/fl^b^fl/fl^	UT Southwestern	Mansuy-Aubert et al., 2015
Mouse: Phox2bCre	UT Southwestern	Liu et al., 2014
Oligonucleotides		
For Primer list see TableS1	This paper	N/A
Software and Algorithms		
Origin	Origin Labs	Origin 2017
SPSS Statistics	IBM	Statistics 24
Real-Time PCR System Software	Applied Biosystems	SDSv2.1
Other		
WD	Envigo/Teklad Diets	TD88137
Normal diet	Envigo/Teklad Diets	Teklad LM-485
Von Frey filaments	North Coast Medical	NC12775–99
Plantar test apparatus (Hargreaves Method)	IITC Life Science	Cat# 390

### CONTACT FOR REAGENT AND RESOURCE SHARING

Further information and requests for resources and reagents should be directed to the Lead Contact, Virginie Mansuy-Aubert (vmansuyaubert@luc.edu).

### EXPERIMENTAL MODEL AND SUBJECT DETAILS

#### Mice Strains

All studies were conducted in accordance to recommendations in the Guide for the Care and Use of Laboratory Animals of the National Institutes of Health and the approval of the Loyola University Chicago and UT Southwestern Medical Center Institutional Animal Care and Use Committee. C57BL/6J (#000664), RiboTag (#011029) were obtained from Jackson laboratory (Maine, USA) and crossed with transgenic mice carrying Cre recombinase driven by a Scn10a promoter (Nav1.8::Cre mice) to generate wildtype and RiboTag+/+:Nav1.8Cre+/– mice. Sensory neuron specific liver x receptor (LXRα and b) knockouts were obtained by crossing LXRα^fl/fl^β^fl/fl^ mice with Nav1.8Cre+/– mice to generate LXRα^fl/fl^β^fl/fl^:Nav1.8Cre+/– which were then crossed with LXRα^fl/fl^β^fl/fl^ to obtain LXRα^fl/fl^β^fl/fl^ (controls) and LXRα^fl/fl^β^fl/fl^:Nav1.8Cre+/–. LXRα and b knockouts in phox2b expressing neurons were obtained by crossing LXRα^fl/fl^β^fl/fl^ mice with phox2bCre+/– mice to generate LXRα^fl/fl^β^fl/fl^:phox2bCre+/– which were then crossed with LXRα^fl/fl^β^fl/fl^ to obtain LXRα^fl/fl^β^fl/fl^ (controls) and LXRα^fl/f^β^fl/fl^:phox2bCre+/–. All mice were housed 4/cage under a 12:12 h light/dark cycle. Mice received either NC (Teklad LM-485) or WD (TD88137, Teklad Diets; 42%kcal from fat, 34% sucrose by weight, and 0.2% cholesterol total) (Envigo, Indiana, USA) for 12 weeks starting at weaning. Body weights (BW) were recorded weekly from weaning. All studies mentioned were done exclusively using male mice to avoid confounding effect of hormones with experimenter blinded to both treatment and genotype.

## METHOD DETAILS

### *In vivo* agonist treatment

WT and RiboTag mice were treated with vehicle or LXR agonist (GW3965; 25mg/kg BW) (Axon Medchem, Virginia, USA) by i.p. twice a week for 3 weeks starting at 8 weeks on WD. Tissues were rapidly dissected and frozen in liquid nitrogen before analysis. Tissue from RiboTag mice were harvested and processed as detailed below. Serum samples were collected before and after treatments.

### Glucose and insulin tolerance tests

Overnight (12hrs) fasted mice were given i.p dose of glucose (1g/kg BW) after measuring fasting glucose levels. Blood glucose levels were then monitored using AlphaTrak glucometer for rodents (Fisher Scientific, Pennsylvania, USA). For insulin tolerance, mice were fasted for 4hrs and given i.p dose of insulin (0.5U/kg BW, Human-R Insulin U100, Lilly) with glucose levels monitored before and after.

### von Frey Mechanical Sensitivity

Mice were investigated for mechanical allodynia using phasic stimulation of von Frey filaments. Briefly, mice were acclimated to the testing chambers for 20 min and were subjected to stimulations with 6 calibrated von Frey filaments (0.16; 0.4; 1; 2; 4; 6; 8 g) (North Coast Medical, California, USA). Filaments were applied for 1 s at 1 s intervals with 5 min break between each set of stimulations, with 6 stimulations per filament. Response frequency for each filament were recorded and 50% threshold was calculated using Hill equation (Origin 2017, OriginLab). A single well-trained investigator took all baseline and experimental measurements for these series of experiments while remaining blinded to the genotype and treatment groups. Mice were evaluated in a quiet room, at a constant temperature and acclimated to the von Frey chambers for at least 20 min, but not restrained in the chamber any longer than necessary to minimize stress and discomfort-induced behavioral variations. Allodynia was characterized in all three behavioral tests as an intense paw withdrawal or licking of the stimulated hind paw (Jolivalt et al., 2016).

### Thermal nociception

Mice were investigated for hyperalgesia using Plantar Test Apparatus (Hargreaves Method) (IITC Life Science, California USA) (Jolivalt et al., 2016). Briefly, after acclimation to testing chambers, tests were performed on the plantar surface of mice by a focused, radiant heat light source with a built-in timer displaying reaction time in seconds. A Humane cutoff time of 20 s was set, at the end of which the heat source shuts off automatically if the animal has not responded, avoiding tissue damage.

### Serum triglycerides, cholesterol, insulin, and leptin measurement

Serum from NC and WD mice either on vehicle or GW3965 were processed for levels of triglycerides (TR22421, Fisher Scientific), cholesterol (TR13421, Fisher Scientific), insulin, and leptin (EMD Millipore, Massachusetts, USA) using manufacturer’s instructions.

### Dorsal Root Ganglia organotypic culture

Juvenile male mice (4–5weeks) were anesthetized with isoflurane before decapitation, and the DRG were quickly removed and cultured on a air-interface membrane (Millipore). Cultures were maintained for a week in standard culture medium (Mansuy-Aubert et al., 2015) replacing every other day in a 37°C and 5% CO_2_ incubator. After an overnight incubation in low serum (2.5%) MEM supplemented with GlutaMAX (2mM), DRG were stimulated with either vehicle or 15μM GW3965 for 24 hr before palmitate treatment (400 μM) for another 24hrs. RNA was extracted using Acturus PicoPure RNA Extraction Kit (Applied Biosystems, California, USA).

### Immunohistochemistry

DRG sections from RiboTag+/+:Nav1.8Cre+/– mice and control mice were used to stain for HA-tag (Biolegend, #901513, California, USA; secondary- goat anti-mouse 488, ab150113, Abcam, Massachusetts, USA) as described before (Sanz et al., 2009).

### Enrichment of transcripts from sensory neurons

DRG from RiboTag+/+:Nav1.8Cre+/– mice were either freshly harvested for RNA isolation or harvested to perform organotypic culture followed by RNA isolation. To isolate RNA associated with HA-tagged ribosomes in sensory neurons, IP followed by mRNA purification following the procedure published by Sanz et al. (Sanz et al., 2009) was used. Briefly, DRG were homogenized in homogenization buffer and supernatant removed after centrifuging at 10,000 g for 10 min at 4°C. 10% of the homogenate was saved (input) for mRNA isolation. Remaining volume was incubated at 4°C with anti-HA antibody (Biolegend, #901513) at 1:150 dilution for 4hrs on a gentle spinner. This is followed by an overnight incubation at 4°C on a gentle spinner with above sample transferred to tube containing magnetic beads (Pierce A/G magnetic beads, California, USA). Supernatant form the samples were collected and beads were washed with high salt buffer, 3 times,10 min each at 4°C on spinner. After final wash, lysis buffer (RNeasy Micro Kit, QIAGEN, Maryland, USA) with β-mercaptoethanol (10μl/ml) was added to elute the mRNA. Total RNA from the IP’ed polysomes was eluted using RNeasy Micro Kit (QIAGEN, California, USA) following manufacturer’s instructions and quantified with Quant-iT RiboGreen RNA Assay kit (Invitrogen, California, USA) and Agilent Bioanalyzer. Quantitative PCR performed on cDNA reverse transcribed from Ribotag mice RNA were normalized to β-actin as previously reported (Nectow et al., 2017; Sanz et al., 2009, 2013; Soden et al., 2016).

### Primary DRG neuronal culture

DRG from juvenile male mice were collected in ice-cold advanced DMEM without any supplementation and axotomized. Axotomized DRG were then transferred to a collagenase A/trypsin mix (1.25mg/ml each) and incubated for 30min. Partially digested DRG were then passed through fire polished glass pipettes followed by 3min spin at 3000 g. After careful removal of supernatant, cells were resuspended in advanced DMEM with 10% FBS and 4mM GlutaMAX, and plated onto a poly-l-lysine coated plates. Neuronal cultures were maintained in a 37°C and 5% CO_2_ incubator for 3–4 days changing above media supplemented with Ara-C (20μM) to inhibit replicative cells every other day before treating the cells to extract RNA as described above.

### Expression profiling of NRs

NR expression in the DRG was done using high throughput method as previous published (Bookout et al., 2006, 2013). Laser capture microdissection was performed on whole lumbar DRG avoiding conjunctive tissues and fibers containing Schwann cells (Leica microsystems, Illinois, USA). DRG were cryosectioned at a thickness of 25μm and thaw-mounted onto saline coated PEN membrane glass slides (Life Technologies, California, USA) and stored at –80°C. Slides were lightly fixed in 75% ethanol followed by thionin staining. Slides were then dehydrated in a graded ethanol series followed by 1 min in xylenes. The Arcturus Veritas Microdissection System (Life Technologies) was used to isolate cells. RNA was extracted using the PicoPure RNA Isolation Kit (Life Technologies) with an additional on-column DNase I treatment to remove genomic DNA (QIAGEN). RNA quality and concentration was evaluated using the Experion Automated Electrophoresis system (Bio-Rad, California, USA).

### Quantitative PCR

For NR profiling, due to the limited amounts of starting material, 0.5 ng of RNA from each isolated DRG sections was converted to cDNA using the High Capacity Reverse Transcription Kit (Life Technologies), and subjected to 14 rounds of pre-amplification using a cocktail of the genes of interest. Primers for the 18S were not included in the pre-amplification assay mix. Prior to pre-amplification, the amplicons were tested to confirm unbiased, uniform amplification. Pre-amplified products were diluted 1/20 and PCR amplified for 50 cycles with TaqMan® Gene Expression Master Mix (Applied Biosystems) with a final concentration of 900 mM of TaqMan® Gene Expression Assays (Life Technologies). Gene expression analysis was performed using a combination of previously published gene assays (Bookout et al., 2006; Fu et al., 2005) and the following inventoried TaqMan® Gene Expression Assays: 18S (Hs99999901_s1), CD36 (Mm01135198_m1), Fabp4 (Mm00445878_m1), Fas (Mm01204974_m1), Tusc5 (Mm03992124_m1). Custom primers were used to differentiate the expression of the PPARγ1 isoform from the PPARγ2 isoform (see Table S1).

qPCR data were analyzed using ABI instrument software SDS2.1. Baseline values of amplification plots were set automatically and threshold values were kept constant to obtain normalized cycle times and linear regression data. Analysis of gene expression was performed using the TaqMan-based efficiency-corrected **Δ**Ct assay. Normalized mRNA levels are expressed as arbitrary units and were obtained by dividing the averaged, efficiency-corrected values for each gene by that for 18S RNA expression in each sample. The resulting values were multiplied by 10^5^ for graphical representation and plotted ± standard deviation from triplicate sample wells. Gene expression was considered to be absent if the Ct value was ≥ 30, low if < 0.025 arbitrary units, moderate if between 0.025–0.25 arbitrary units, and high if > 0.25 arbitrary units as described and published before (Bookout et al., 2006, 2013). For all other genes of interest, qPCR was performed using Sybr green-based assay (Roche, Indiana, USA) using IDT primers (IDT technologies, Iowa, USA). See Table S1 for primer list. 18 s (β-actin, for RiboTag:Nav1.8 IP’ed mRNA) was used to normalize data and quantification was done using **ΔΔ**CT method with vehicle treated group’s mean value set at 100%.

### Western Blotting

Whole DRG protein isolation and western blotting was performed as described before (Gavini et al., 2014) and processed for actin and CHOP (abcam, ab8226, ab11419 respectively) at supplier recommended dilutions. For HA, IP was performed using anti-HA (Biolegend, #901513) and membrane was probed with rat anti-HA-peroxidase (Sigma-Aldrich, #12013819001, Missouri, USA).

## QUANTIFICATION AND STATISTICAL ANALYSIS

All data are represented as mean ± SEM. Analyses were done using IBM SPSS Statistics 24. For single group comparisons either a 1-or 2-tailed t test was used as appropriate and multiple comparisons were performed using ANOVA. For repeated-measures, 2-way ANOVA was used and p value less than 0.05 was considered significant.

## Supplementary Material

1

2

## Highlights

- LXRs, transcriptionally active in the dorsal root ganglia, regulate *Abca1* and *lpcat3*
- LXR agonist protects the dorsal root ganglia from palmitate-induced ER stress
- LXR agonist treatment delays obesity-induced allodynia in mice
- LXRs agonist delays western diet-induced ER stress in sensory neurons *in vivo*
