# Supplementary material for: Liver X Receptors Protect Dorsal Root Ganglia from Obesity-Induced Endoplasmic Reticulum Stress and Mechanical Allodynia
Source: Cell Rep. Author manuscript; Available in PMC 2020 Dec 11. (PMC7732131; doi:10.1016/j.celrep.2018.09.046)
Supplement: 1 [file NIHMS1510166-supplement-1.pdf]

Cell Reports, Volume *25*

Supplemental Information

Liver X Receptors Protect Dorsal Root Ganglia from Obesity-Induced Endoplasmic Reticulum Stress and Mechanical Allodynia

Chaitanya K. Gavini, Angie L. Bookout, Raiza Bonomo, Laurent Gautron, Syann Lee, and Virginie Mansuy-Aubert

Figure S1

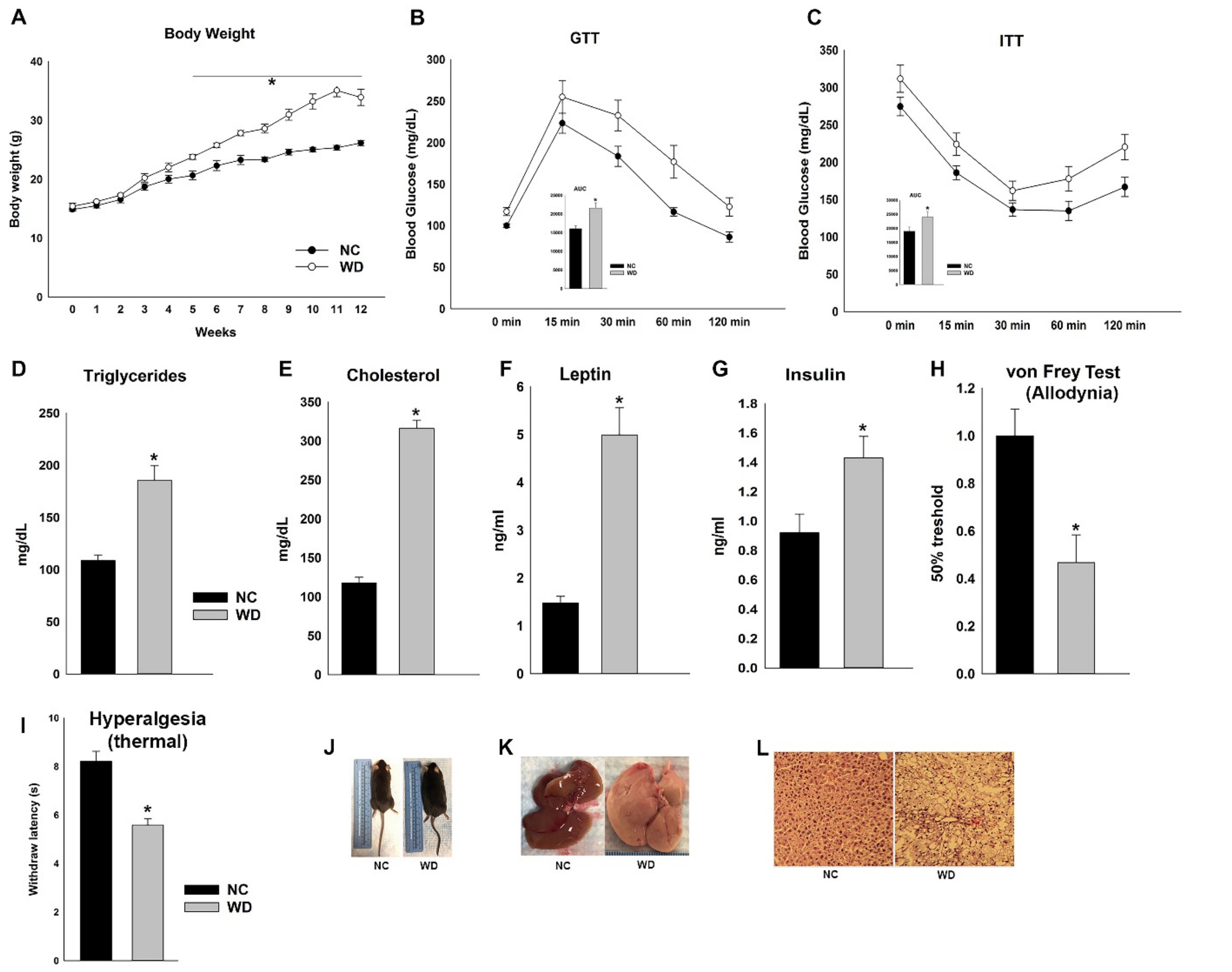

Figure S1: Western diet induces obesity, lipid accumulation, and allodynia. Related to Figure 2. (A) Body weight of mice on normal diet (NC) and western diet (WD) over 12 weeks. (B) Intraperitoneal glucose tolerance test (GTT) of NC, and, WD-fed mice. (C) Intraperitoneal insulin tolerance test of NC, and, WD-fed mice. Levels of serum triglycerides (D), cholesterol (E), leptin (F), and insulin (G) in WD, and NC-fed mice at end of 12 weeks. (H) Von Frey test to assess allodynia, relative threshold values represented with mean 50% threshold of NC mice as 1. (I) Thermal sensitivity test (Hargreaves method) in NC and WD-fed mice. (J) Mice fed on either normal diet (NC) or western diet (WD) for 12 weeks. (K) Representative images of livers of NC and WD mice after 12 week of diet. (L) H and E staining on liver sections of NC and WD mice. (All values are mean±S.E.M; n=8/group; *p<0.05 with respect to NC controls).

Figure S2

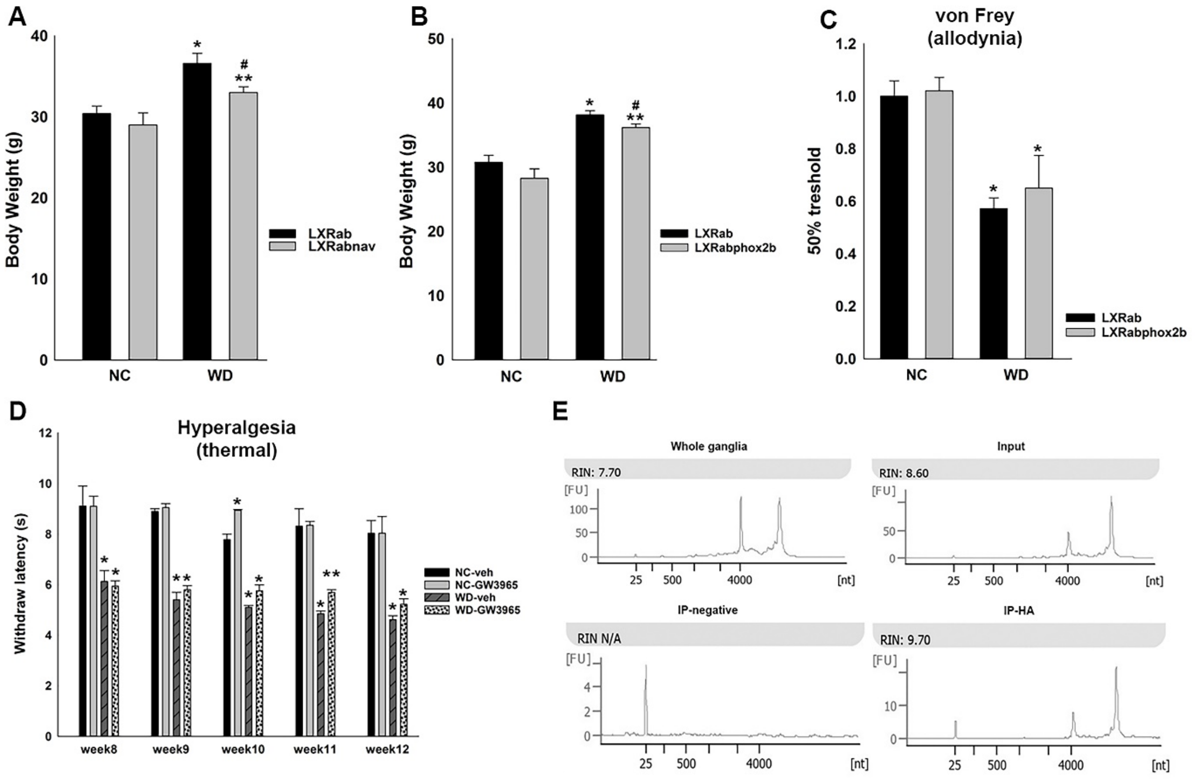

Figure S2: Related to Figure 3. (A) Body weights of LXRab (control) and sensory neuron specific LXRab knockout (LXRabnav (mice lacking LXR in Nav1.8 expressing neurons)) after 12 weeks on normal (NC) and western diet (WD); (n=8/group) *$p<0.05$ compared to LXRab NC, **$p<0.05$ compared to LXRabnav NC, #$p<0.05$ compared to LXRab WD mice. (B) Body weights of LXRab (control) and LXRabPhox2b (mice lacking LXR in Phox2B) expressing neurons after 12 weeks on normal (NC) and western diet (WD); (n=8/group) *$p<0.05$ compared to LXRab NC, **$p<0.05$ compared to LXRabphox2b NC, #$p<0.05$ compared to LXRab WD mice. (C) von Frey test to assess sensitivity of LXRab and LXRabphox2b mice on either diet to innocuous stimuli (n=8/group), *$p<0.05$ compared to LXRab NC. (D) Thermal nociception test to assess thermal sensitivity of mice on either diet treated with LXR agonist (e.g Week 8 = baseline, 8 weeks on WD; Week 9 = 1 week after agonist admission, 9 weeks on WD) (n=8/group) *$p<0.05$ compared to NC-veh, **$p<0.05$ compared to WD-veh. (E) Bioanalyzer trace of mRNA from DRG samples of whole ganglia, RiboTag-Nav1.8-Cre (input, IP-negative control, and IP-HA). All values are mean±S.E.M.

Table S1: qPCR primer list. Related to STAR Methods

	Forward (5'-3')	Reverse (5'-3')
18s	AGGACCGCGGTTCTATTTTGTTGG	ATGCTTTCGCTCTGGTCCGTCTTG
CHOP	CCACCACACCTGAAAGCAGAA	AGGTGAAAGGCAGGGACTCA
XBP1	TGGCCGGGTCTGCTGAGTCCG	GTCCATGGGAAGATGTTCTGG
sXBP1	CTGAGTCCGAATCAGGTGCAG	GTCCATGGGAAGATGTTCTGG
usXBP1	CAGCACTCAGACTATGTGCA	GTCCATGGGAAGATGTTCTGG
ATF4	GGGTTCTGTCTTCCACTCCA	AAGCAGCAGAGTCAGGCTTTC
LPCAT3	TCTGGGGCAAATTTGTGCTG	AGCCACACTTTCATGTTGGC
Nav1.8	TGCTGCAAAGTGAACACCAG	ATGCGGTAACAGGTTTTGCG
GFAP	TGCTGGAGGGCGAAGAAA	CGGATCTGGAGGTTGGAGAA
PPARγ1	GCGGCTGAGAAATCACGTT	TCAGTGGTTCACCGCTTCTT
PPARγ2	CACCAGTGTGAATTACAGCAAATC	ACAGGAGAATCTCCCAGAGTTC
Abca1	CGTTTCCGGGAAGTGTCCTA	GCTAGAGATGACAAGGAGGATGGA
PV	GACACCACCTGTAGGGAGGA	AGTACCAAGCAGGCAGGAGA
Actin	ACCTTCTACAATGAGCTGCG	CTGGATGGCTACGTACATGG
LXRa	AGGAGTGTCGACTTCGCAAA	CTCTTCTTGCCGCTTCAGTTT
LXRb	CTCCCACCCACGCTTACAC	GCCCTAACCTCTCTCCACTCA
ABCG1	GCTGTGCGTTTTGTGCTGTT	TGCAGCTCCAATCAGTAGTCCTAA
ATF3	CCAGGTCTCTGCCTCAGAAG	CATCTCCAGGGGTCTGTTGT
Apo E	GCAGGCGGAGATCTTCCA	CCACTGGCGATGCATGTC
Srebp1f	TACTCGAGCCTGCCTTCAG	TAGATGGTGGCTGCTGAGTG
Gap43	TTTTGATCTGGTGCGTGTGG	ACGGAACATTGCACACACAC
